# Prebiotic Potential of Cereal Components

**DOI:** 10.3390/foods10102338

**Published:** 2021-09-30

**Authors:** Reihane Abdi, Iris J. Joye

**Affiliations:** Department of Food Science, Ontario Agriculture College, University of Guelph, Guelph, ON N1G 2W1, Canada; rabdi@uoguelph.ca

**Keywords:** wheat, dietary fiber, arabinoxylans, β-glucans, fructans, fructooligosaccharides, resistant starch, immature wheat grain

## Abstract

One type of functional food that has been receiving much attention is food rich in prebiotics. The old but still valid definition of prebiotics defines them as non-digestible food components that selectively stimulate the growth and/or activity of the beneficial bacteria in the colon and, as a result, improve the host health. Cereals, as one of the main components in the human diet, contain substantial levels of dietary fiber with probable prebiotic potential. In addition, dietary fiber, particularly soluble dietary fiber, has recently emerged as a promising natural highly functional food ingredient in food production. This review focuses on the prebiotic potential of cereal dietary fiber types and covers the achievements and developments regarding its isolation. First, the probiotic and prebiotic concepts will be discussed. Next, different components of dietary fiber and their effect on the host bacteria through in vitro and/or in vivo studies will be reviewed. In a last part, this paper also discusses means of boosting the prebiotic properties of cereal components and innovative strategies for the extraction of cereal dietary fiber. The review focuses on wheat as a leading cereal crop that is widely and intensely used throughout the world in food production.

## 1. Introduction

The emerging interest of consumers in foods having high nutritional value and beneficial health implications has resulted in a remarkable boost of probiotic and prebiotic food product development. In this context, cereal-based products have received substantial attention. Cereals and cereal constituents can be used as fermentable substrates for probiotic microorganisms, hence providing prebiotic effects [1]. In fact, aside from boosting several beneficial physiological effects by themselves, certain cereal components can also selectively prompt the growth and/or activity of the bacteria present in the colon; and therefore, improve the host health [2,3].

Probiotics are defined as “live microorganisms which when administered in adequate amounts confer health benefits to the host” [3]. These living salubrious bacteria should pass unharmed across the upper gastrointestinal tract (GIT) and eventually populate and thrive in the colon [4]. Thriving of probiotic bacteria in the digestive tract can be promoted by prebiotics. Prebiotics were first defined as “non-digestible food ingredients that beneficially affect the host by selectively stimulating the growth and/or activity of one or a limited number of beneficial bacteria in the colon, and thus improve host health” [5]. Since this first definition, the concept of prebiotics was amended by the International Scientific Association of Probiotics and Prebiotics (ISAPP) in December 2016 as “a substrate that is selectively utilized by host microorganisms conferring a health benefit” [6]. Combining probiotics and prebiotics exerts a synergistic effect on the host health [3].

**Figure 1:**
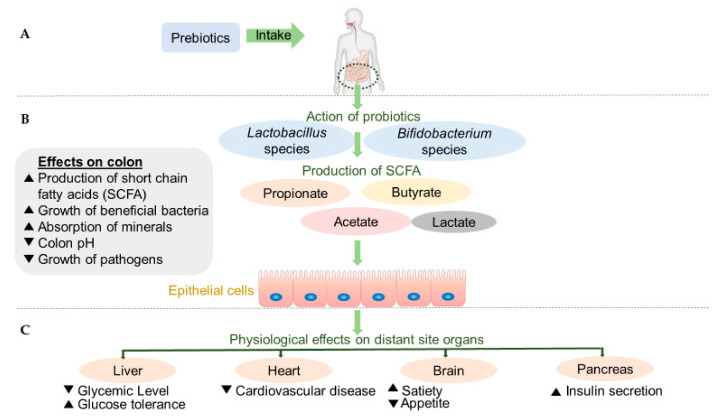
Effect of prebiotics on human health. (**A**) Consumption of prebiotics, (**B**) Direct effects of prebiotics on colon, (**C**) Indirect effects of prebiotics on other organs. Corresponding symbols ▲ and ▼ stand for “increase” and “decrease”, respectively. Based on Farias et al. [6].

Cereals are well-known sources of dietary fiber (DF). DF is often classified according to its water-solubility in water-soluble (e.g., β-glucans, water-extractable arabinoxylans (AX), arabinoxylan oligosaccharides (AXOS), galacto- and fructooligosaccharides (GOS and FOS)) and water-insoluble fiber (e.g., water-unextractable AX, resistant starch (RS), cellulose, lignin). Water-soluble DF, in particular, have been attracting interest as prebiotic ingredients [7,8]. These carbohydrates resist digestion in the upper GIT, which makes that they can be used as a carbon and energy source for bacteria in the gut [9] (Figure 1). The carbohydrates are eventually fermented by beneficial bacteria in the gut such as bifidobacteria and lactobacilli species (Figure 1), promoting their growth and the production of short-chain fatty acids (SCFA). Butyrate, acetate and propionate are the most important SCFA that provide metabolic energy for the host and result in acidification of the bowel content [8,10]. Moreover, prebiotics have the potential to impact not only the bacterial population in the GIT, but also to have an effect on other organs (Figure 1). SCFA reach the blood circulation by diffusing through the epithelial cells and, as such, affect distant organs and systems (Figure 1) [11]. In fact, production of these acids is associated with the amelioration of some physiological health aspects such as improvement of mineral absorption, lowering colon cancer risk, regulation of glucose and lipid metabolism along with improving intestinal function [6]. Furthermore, since production of SCFA results in lower colonic pH, the growth of potential pathogens in the colon is reduced [8].

In this review paper, the current knowledge on prebiotic efficacy of cereal components and the innovative technologies for their isolation, with the focus on wheat as one of the world’s most widely grown and diverse crops, are discussed.

## 2. Dietary Fiber

Cereals are the main source of DF in the human diet [12]. Indeed, DF is one of the prime constituents of cereals and cereal products and has a well-described health-promoting functionality [13]. The most common definition of DF proposed by the Codex Alimentarius 2009 states that “DF consists of carbohydrate polymers with 10 or more monomeric units, which are not hydrolyzed by the endogenous enzymes in the small intestine”. Nonetheless, national authorities are allowed to include “carbohydrates of 3–9 monomeric units” in this definition [12]. The type and (absolute and relative) content of DF types vary largely between cereals (Table 1).

Based on water-solubility, DF can be divided into two classes. Water-soluble (sometimes referred to as water-extractable) and water-insoluble (or water-unextractable) fibers provide distinct physiological effects [7]. The most important cereal water-soluble DF are β-glucan and water-extractable arabinoxylans (WE-AX). Water-insoluble cereal fiber comprises mainly cellulose and water-unextractable arabinoxylans (WU-AX) [7,14]. Water-soluble DF are generally less resistant to colonic fermentation than water-insoluble DF and present a potential prebiotic effect [8,15]. The prebiotic potential and physiological effects of DF indeed depend on its physicochemical properties, affected by e.g., the degree of polymerization (DP), chemical structure (e.g., side chain presence and distribution), extent of intermolecular cross-linking and the degree of degradation in the bowel [8].

The major wheat DF types are arabinoxylan (AX), mixed-linked (1-3),(1-4)-β-d-glucan (β-glucan), cellulose and lignin [8,13]. Among the cereal DF components with well-described functional properties, β-glucan, fructans (FOS and inulin) and GOS have well-proven prebiotic activity [3,8,12,16,17,18]. Prebiotic activity of wheat AX has also been reported [12,16].

DF in cereals are predominantly molecules found in the walls of cells in the cereal kernels, with the exception of arabinogalactan-peptides (AGP) and fructans [19,20,21]. Indeed, the main DF found in wheat flour stem from these endosperm cell walls, and 2 to 3% of the dry wheat flour weight is DF. The most important DF in wheat flour are cell wall polysaccharides including AX (~70 wt% of the total cell wall components) and β-glucan (~20 wt%). Minor cell wall polysaccharides of wheat flour comprise glucomannan (~7 wt%) and cellulose (~2 wt%) [12,19]. Additionally, some of the arabinose and galactose found in wheat flour stem from AGP, which constitute up to 0.5% of the dry weight of white wheat flour. Whole wheat kernels can contain up to 15.5 wt% DF. Especially the pericarp and aleurone layers are DF rich [12]. In cereals, the amount of DF typically sharply declines from the outer pericarp to the endosperm. AX, however, the main constituent of cell wall material in endosperm, shows a less sharp decrease from outer kernel layers to inner endosperm [7].

### 2.1. Arabinoxylans

**Figure 2:**
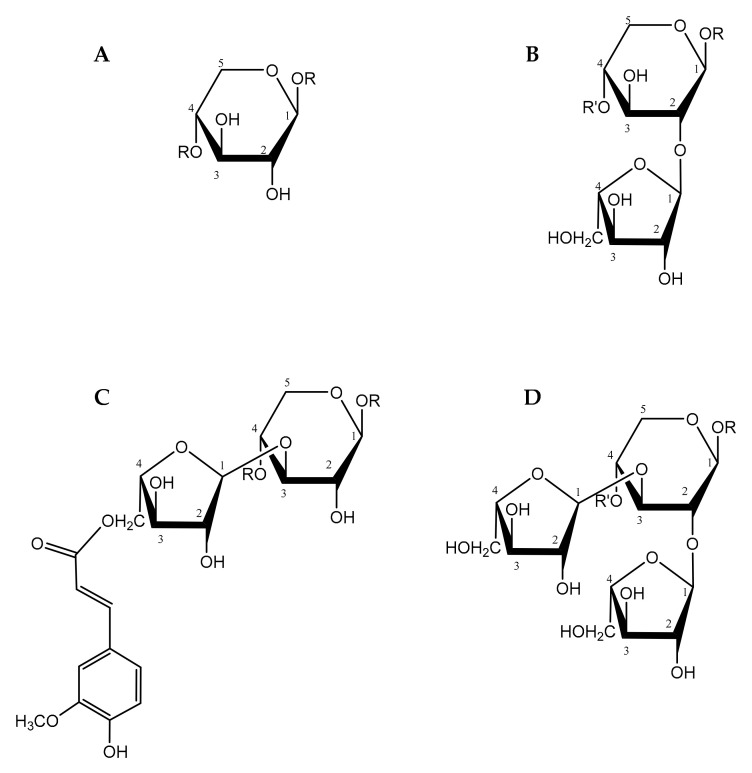
The general building units of arabinoxylan (AX) molecules. (**A**) Unsubstituted xylose unit; (**B**) Xylose residue substituted at C(O)-2 with an arabinose residue; (**C**) Xylose residue substituted at C(O)-3 with an arabinose residue, a ferulic acid moiety esterified to the arabinose at C(O)-5; and (**D**) disubstituted xylose unit at C(O)-2 and C(O)-3 with arabinose units.

AX are part of the main non-starch polysaccharides (NSP) in cereal cell walls [22]. The AX backbone is composed of β1→4 linked xylopyranose units; carrying arabinofuranose moieties at the C-2 and/or C-3 positions of the xylopyranose units (α1→2 and/or α1→3 linkages) [22]. Moreover, ferulic acid can also be esterified to the hydroxyl group at the C-5 position of the arabinose substituents (Figure 2) [16,22]. AX are categorized into WE-AX and WU-AX, based on their solubility/extractability in water [22,23]. The molecular weight and degree of diferulate crosslinking are determining factors for the water extractability of AX [23]. WE-AX and WU-AX share a general structure. It is speculated that WE-AX serves as a building block for WU-AX. The poor water extractability associated to a large fraction of cereal AX stems from the high molecualr weight of the AX molecules. Evidently, diferulate cross-links between AX molecules result in even larger molecular weights [24].

In wheat, AX constitute ~50 wt% of the DF fraction and are mainly found in the bran and aleurone fractions [14]. AX account for around 85 wt% of NSP in the entire wheat kernel [16]. In wheat bran, AX make up ~65 wt% of the NSP; while in wheat endosperm, ~88 wt% of the NSP is AX [14]. The aleurone layer around the wheat endosperm broadly consists of 60-70 wt% AX. In common wheat, WE-AX and total AX contents in kernels were found to be 0.7 and 6.7 wt%, respectively [25]. The hardness of kernels was reported to be positively correlated with the total AX content in wheat flour [13]. Moreover, WE-AX content in soft white winter wheat cultivars was slightly higher than what was reported for soft white spring cultivars [26].

Although AX are potential prebiotics and have been found to be hydrolyzed in the large intestine by bacterial hydrolytic enzymes, such as xylanases and arabinofuranosidases, the physiological effects of AX are largely unknown [14]. Nonetheless, it was reported that the biological function of AX such as their prebiotic activity is related to the chemical AX structure [22,23]. In wheat, for instance, it was found that the type of cultivar and the tissue from which AX are extracted affect the chemical structure of AX and, as such, their prebiotic activity [22]. In a study by Paesani et al. [22], the molecular size distribution of the WE-AX extracted from whole wheat flour (Argentinian hard and soft varieties) was found to affect the potential prebiotic activity. While all tested samples had a similar A/X ratio for WE-AX, the molecular size of WE-AX in hard wheat was reported to be higher than what was found in soft wheat. WE-AX in the tested hard wheat also showed a higher prebiotic activity than WE-AX in the tested soft wheat samples. Both hard and soft wheat WE-AX displayed their potential prebiotic effect through increasing the growth of beneficial bacteria, i.e., lactobacilli and bifidobacteria, in in vitro and in vivo tests. The prebiotic efficacy of the extracts was also shown in vivo through the decrease in the clostridia count in the gut microorganism profile and an increased production of SCFA in the bowel. Among the SCFA produced during fermentation of AX, acetate was the most dominant acid [22]. This is in line with the findings in a study by Rumpagaporn et al. [27] that focused on the in vitro fermentation of AX extracted from the bran of wheat, rice, corn and sorghum. Ultimately, a significant correlation between the prebiotic potential assessed in in vivo and in vitro tests was found with molecular size of AX, thus allowing the prediction of the potential prebiotic effect of WE-AX present in whole wheat flour [22]. In contrast, Hughes et al. [28] reported that in a small-scale human fecal batch cultures, the prebiotic effect (selectivity for lactobacilli and bifidobacteria) of commercial wheat AX fractions differing in molecular mass increases as the molecular mass decreases. They showed a significant increase in total SCFA production, and particularly butyrate, as a result of AX fermentation [28].

Studies focusing on other structural features of AX showed that ferulic acid substitution and branching patterns also play a major role in the fermentation of AX. Indeed, a negative correlation was reported between the number of esterified ferulic acid groups and AX fermentability. Hopkins et al. [29], e.g., reported that the fermentation rate by human fecal microbiota of feruloylated AX is much lower than what was found for non-ferulated AX; although both show a similar SCFA profile. Moreover, hydrolytic degradation of AX by bacterial enzymes is slowed down by the side chains of AX in comparison to the linear unsubstituted chain [30]. In the study by Rumpagaporn et al. [27], it was clearly reported that for highly branched AX polymers that had high degrees of substitution, the effect of molecular size, A/X ratio and degree of substitution did not relate to the fermentation rate patterns [27]. The rate of fermentation was rather affected by the nature of the side branches. A higher terminal xylose content in the side branches of corn and wheat AX molecules slowed down the fermentation rate [27]. It is noteworthy that the structure of AX molecules differs based on their location in the cereal kernel and also varies among different cereal grains. In another study with pigs, rye WE-AX with a low degree of xylan backbone substitution were readily fermented, whereas rye AX with comparably high mono- and disubstitution, terminal xylose, and non-terminal arabinose were more difficult to degrade. It was reported that the fermentation of wheat aleurone AX, i.e., lightly branched AX were more readily fermented than the heavily branched AX [23]. Although these correlations have been the center of attention in recent studies, the high degree of diversity in structure of AX molecules makes it complicated to construct straightforward structure-effect relationships. In this regard, the relationship between the AX structure and the physiological effects, i.e., SCFA production and promotion of the growth of colonic bacteria in vivo and in vitro, remains to be fully elucidated. However, the above also shows that it is theoretically possible to compose and select a specific AX subpopulation that has customizable fermentation rate characteristics and, hence, prebiotic effects.

### 2.2. Arabinogalactan-Peptides

AGP are NSP consisting of arabinose and galactose building units. In many plant species, the arabinogalactan oligosaccharide is covalently linked with a peptide, hence, forming AGP [12,31]. The hydroxyproline-rich peptide core of AGP is linked to arabinose and galactose rich oligosaccharide units. Wheat AGP make up ~0.5% of the dry weight of the endosperm [12,20]. Slightly lower contents of AGP were reported in flours of other cereal grains: triticale (0.32 wt%), barley and spelt (0.28 wt%) and rye (0.21 wt%) [20]. In white wheat flour, AGP comprise a 15-residue amino acid peptide containing three hydroxyproline residues which are *o*-glycosylated with branched arabinogalactan chains [12]. Fincher and Stone [32] reported that the arabinogalactan oligosaccharides cover approximately 90% of the molecular mass of the wheat flour AGP with an arabinose to galactose (A/G) ratio of 0.69. Other studies also reported similar A/G ratios in AGP isolated from wheat flour. For instance, a comparatively consistent A/G ratio (0.66–0.75) was found for AGP isolated from eight Canadian wheat varieties [33].

Recent studies have detected AGP in vacuoles or the cytoplasm of wheat kernel cells. However, there is a lack of information on the physiological effects of AGP both in grain development and human health [12]. The potential prebiotic activity of wheat AGP was very recently demonstrated by the selective promotion of *Bifidobacterium* genus and production of SCFA (mostly acetate) during in vitro fermentation, using fecal samples [12]. AGP underwent a slower bacterial fermentation than what was found for FOS, demonstrating that AGP endure better the conditions in the distal regions of the colon. A slower rate can be advantageous for health as it allows the beneficial bacteria to reach the more distal regions of the colon. However, studies focusing on the fermentation of a mixture of AGP and WE-AX, with the same ratio as is present in white wheat flour, showed that the fermentation rate was increased relative to the fermentation rate that was found for the individual substrates and the mixture’s fermentation rate was more comparable to that determined for FOS. Therefore, it was concluded that a mixture of AGP and AX, as fermentable carbohydrates, may act synergistically and can likely be more efficiently used as prebiotic than the individual substrates. However, these results need to be substantiated in in vivo human studies to further elucidate the interplay of AGP and AX in fermentation processes in the colon [12].

### 2.3. β-Glucan

**Figure 3:**
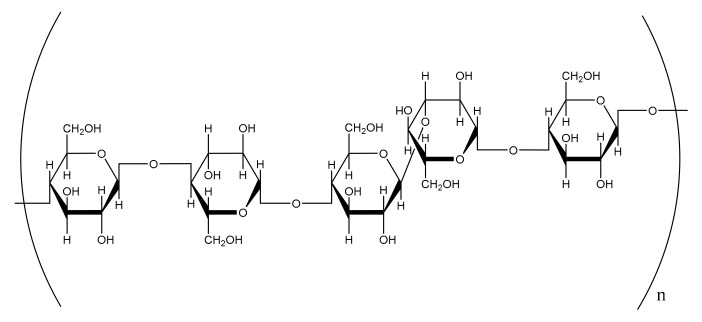
Structure of cereal β-glucan.

β-glucan is one of the major subclasses of the cereal DF family, and is a linear polysaccharide found in the cell walls in the endosperm [7]. This NSP is built of glucose units that are linked by β-(1→4) and/or (1→3) linkages (Figure 3).

Of all cereal grains, barley and oat grains contain the highest β-glucan levels, i.e., 3–11% and 3–7% on dry basis (d.b.), respectively [7]. However, the level of β-glucan can vary dramatically between varieties. β-glucan is customarily located in the cell walls in endosperm of barley, oats, rye (2% d.b.) and wheat (≤0.5% d.b.) [7]. The crease area particularly in wheat, and conceivably also in other cereal grains, was found to carry a noticeable level of β-glucan [7]. Although wheat is not considered a rich source of β-glucan, the physical kernel characteristics allowed the development of a pearling technology to separate the outer layers from the endosperm, hence, obtaining β-glucan enriched wheat-derived tissue [7].

β-glucans have regained attention in the food industry over the past few years due to their unique functional properties (e.g., as thickening, stabilizing and gelling agents), and biological effects (e.g., as an anticipated prebiotic), making these DF an excellent choice as functional ingredients for the food industry. The molecular weight of cereal β-glucans is highly variable and this could be a key parameter determining microbial growth in the colon and, hence, in their functionality as prebiotic compounds [34]. For example, hydrolysates of β-glucans with molecular weights of 137, 150 and 172 kDa were reported to promote the growth of *Bacteriodes* and *Prevotella* species in in vitro conditions. Larger size hydrolysates of 230 and 243 kDa, conversely, did not significantly increase the growth of the tested bacteria [35].

Several examples are worth citing with regard to prebiotic claims for oat and barley sourced β-glucan that selectively supports the growth of probiotics, mostly lactobacilli and bifidobacteria in in vivo and in vitro conditions. Oat β-glucan hydrolysates have been reported to prompt the growth of *Lactobacillus rhamnosus* GG in vitro [36]. In a study by Vasiljevic et al. [37], addition of oat and barley β-glucans to probiotic yogurt inhibited the proteolytic activity of the probiotic strain, *Bifidobacterium animalis* ssp. lactis Bb-12TM, and ameliorated viability and also stability of the strain. However, both oat and barley β-glucans resulted in a suppressed proteolysis.

Several studies have been performed focusing on the physiological effects, such as anti-hypercholesterolemic, anti-oxidant, and anti-bacterial effects of oligosaccharides derived from barley β-glucan [38]. A recent study by Lee et al. [39] reported the prebiotic activity of β-gluco-oligosaccharides (β-GOS) derived from barley β-glucan. These oligosacharides consist of 3-*O*-cellobiosyl-d-glucose and 3-*O*-cellotriosyl-d-glucose and are able to selectively modulate the growth and antimicrobial activity of tested probiotics in in vitro conditions. Although most of the tested pathogens slightly hydrolyzed these β-glucan derivatives, the derivatives showed no contribution to the procreation of pathogens. Therefore, there is a possibility for these compounds to negate the negative effects of pathogens. Production of bacteriocins or bacteriocin-like substances by probiotic strains of *Lactococcus lactis* subsp. lactis, *Lactobacillus reuteri*, and *Pediococcus acidilactici*, was increased in the presence of β-GOS as the sole carbon source. These results indicated that combining β-GOS and β-GOS-fermenting probiotics can function as a highly efficient symbiotic system for nutritional and industrial applications [39].

It is noteworthy that although several studies reported on the prebiotic activity of cereal β-glucans, these polysaccharides are usually consumed in combination with other components. Particularly in wheat products e.g., β-glucan is often present together with AX in a ratio of ~1:3. In vitro fermentation studies of wheat AX and barley β-glucan containing foods that are daily consumed by humans, showed that the fermentation of AX alone notably increased the activity of bifidobacteria and resulted in production of SCFA, mostly acetate, while consumption of β-glucan alone did not affect the prebiotic activity. When focusing on the production of total SCFA and the total bacterial count (including beneficial *Bifidobacterium* and *Clostridium coccoides*/*Eubacterium* groups), the maximum prebiotic activity was reported for a mixture of AX and β-glucan in a ratio of 3:1. This ratio is in line with the AX/β-glucan ratio naturally found in wheat flour. Wheat has functioned as a major source of DF in the human diet for thousands of years and it is, hence, possible that the population of bacteria in the human GIT adjusted itself to the diet’s regular DF composition in order to magnify the prebiotic effect [19].

### 2.4. Fructans

Cereal grains and their products are undoubtedly the main source of fructan in our daily diet. Although the high intake of cereal products may result in an immense effect of fructan on colon health, high consumption levels of fructan (e.g., up to 20 g/day) could also provoke mild to severe bloating and flatulence, depending on the consumers sensitivity and overall health [40,41]. A selection of some reports on fructan levels in wholegrain cereals is outlined in Table 2.

The data shows that rye contains the highest fructan level; while oat grains generally have the lowest level of fructan among the studied cereal grains. Macleod and Preece [48] also reported an increase in fructan content in the order, oat, barley, wheat and rye. The fructan concentration in wheat flour is slightly lower than what is found for the wholegrain; and the bran fractions appear to possess a higher amount of fructan than the endosperm. For example, the fructan levels reported for three different wheat flour samples (1.4–1.7%) were certainly lower than what was found in the related fractions of bran (3.4–4.0%) [21].

It is obvious that the immature kernels of most cereal grains possess a significantly higher level of fructan in comparison to the mature kernels [49]. As an example, in immature grains of wheat, rye, barley and triticale, the amount of fructan was reported to approximate 23.7–39.0 g/100 g dry meal [50]. With respect to wheat kernels, accumulation of fructan has been reported to occur in the cell division and expansion phase [40,51]. Filling of the kernels is initiated 14 days after flowering, and is characterized by a swift accumulation of starch and a reduction of the moisture content and level of water-soluble sugars [52]. The changes in level of fructan in developing grains will be discussed in more detail later.

**Figure 4:**
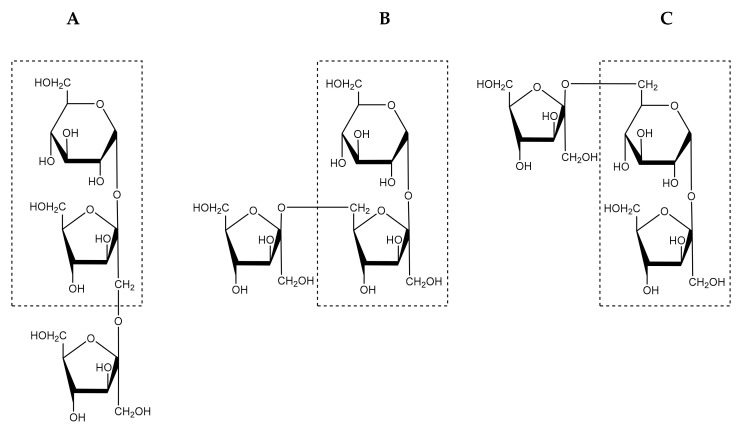
Structure of trisaccharides building fructan structure: (**A**), 1-kestotriose; (**B**), 6-kestotriose; and (**C**), 6G-kestotriose. The sucrose core is shown in the rectangle box. Based on Verspreet et al. [40].

**Figure 5:**
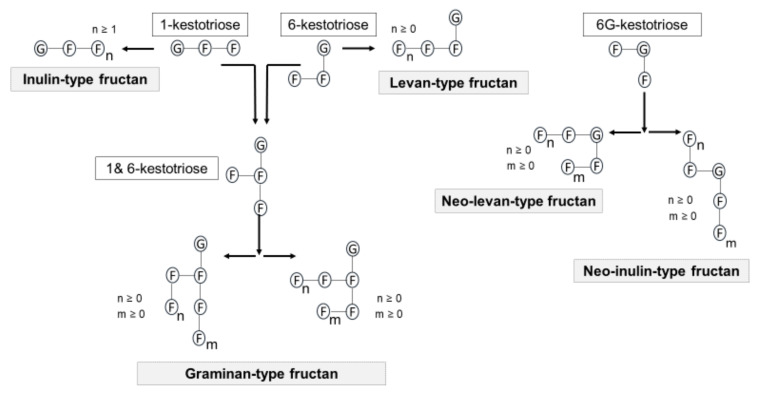
Three core trisaccharides are the main building blocks for different types of fructans. G and F represent glucose and fructose residues, respectively. Based on Verspreet et al. [40].

Fructan is mainly composed of fructose units that are linked through either β (2→1) or β (2→6) fructose-fructosyl linkages. Fructan production is initiated by the addition of fructose to sucrose [40]. Based on the site of the fructose addition, three core trisaccharides i.e., 1-kestotriose, 6-kestotriose or 6G-kestotriose, are generated (Figure 4). These fructan trisaccharides serve as the main building blocks for five types of fructans. These five fructan types are often referred to as the inulin-type, levan-type, graminans, neo-inulin type and neo-levan type (Figure 5). In inulin-type fructans, 1-kestotriose initiates the synthesis of a linear structure composed mainly of fructosyl units linked by β (2→1) linkages. With regards to health benefits, inulin, oligofructose and FOS are the best known inulin-type fructans [53].

Profound in vitro and in vivo research studies proved the health promoting potential of these type of fructans and acknowledged them as prebiotics [53,54]. Fructans are resistant to enzymatic reactions taking place in the upper GIT; whereas they are readily hydrolyzed by the specific hydrolases of some bacterial species in the lower GIT and, consequently, fermented there with the production of SCFA and/or gases [2]. Positive health effects of inulin-type fructans are not only limited to their effect on the colonic bacteria as recent evidence pointed to a direct immunoregulatory impact of these fructans on the innate immune system. In fact, they stimulate the Toll-like receptors, responsible for activation of cell responses; and as a result, seem to trigger protective effects against oxidation ex vivo [55].

### 2.5. Fructooligosaccharides

FOS are oligosaccharides of fructose with low molecular weight (DP ≤ 10) which have received a great deal of attention for their prebiotic properties. FOS are stored in the tissues of several plants as carbohydrate source or as osmoregulators [52]. In comparison to the other natural sources of FOS such as garlic (0.6% d.b.), bananas (0.3% d.b.), onions (0.23% d.b.) and tomatoes (0.15% d.b.), the level of FOS during the grain filling stage is very high in grains. Particularly durum wheat has FOS levels that are 10 times higher than those found in other plants [52]. In spring wheat, FOS content was reported to compose 27% of the kernel dry matter at anthesis, i.e., 0.3 mg per kernel [56].

Numerous studies have reported on the relationship between FOS level and kernel development in grains. The high content of FOS was reported during the filling of grain and particularly in the milky phase, 14–21 days after anthesis [40,57]. At this physiological stage, the grain is immature and still green [49]. Hereafter, the concentration of FOS swiftly reduces. In comparison to mature wheat kernels, immature wheat grain (IWG) contains higher fiber and soluble sugar levels, while the starch amount is low [57]. In fact, the onward decline of FOS and water-soluble sugar levels coupled with the continuous increase in starch content during grain filling, suggests that the high water-soluble sugar levels originate from FOS hydrolysis and are employed for the synthesis of starch. The branched molecules of FOS in IWG possess both β 2→1 and β 2→6 linkages, and have a low DP [57].

IWG as a natural FOS source, have been the focus of both in vivo and in vitro studies on survival of intestinal bacteria. IWG were used by probiotic bacteria over a 14 days of cold storage period in probiotic yogurts, and an increase in mean titratable acidity in the IWG-containing probiotic yogurts was observed. The effect of IWG was observed to be probiotic strain-dependent. For example, the supplementation of yogurt with IWG increased the number of *Lactobacillus acidophilus* NCFM and *Lactobacillus acidophilus* 20079 at the end of the shelf-life of the yogurt; whereas, presence of IWG had no significant effect on *Lactobacillus casei* 431 count [49].

In an in vitro study using an ileostomy model system, bifidobacteria and lactic acid bacteria counts were reported to be significantly higher when IWG-enriched biscuits were tested than control biscuits, corroborating the prebiotic impact of IWG [57]. The prebiotic effect of IWG was mainly related to FOS in the grains, although the contribution of other DF components present in these grains was also worth considering [57]. The in vitro results confirmed the efficiency of IWG as a prebiotic ingredient in formulating functional prebiotic foods. In vivo data suggested that IWG also impacts gastric emptying and satiety [57].

IWG seem to be a great raw material for developing new functional foods; as it is not only a natural source of FOS, but also has other nutritional properties such as similar protein content to mature wheat, significant amount of vitamin C and antioxidant activity [57]. In addition, durum wheat is also strongly suggested as an efficient and alluring source of FOS in large-scale formulation of functional food products [52]. Casiraghi et al. [58] considered IWG as a notable ingredient for making functional products naturally possessing high content of FOS and fiber. They reported that enriching pasta with IWG results in promoting the nutritional quality through an increase of the fiber level. Their results showed that the glycemic index of IWG-enriched pasta is not significantly different from the commercial pasta made from enriched inulin or 100% wholemeal.

### 2.6. Resistant Starch

The majority of the starch found in hydrothermally processed cereal grains is readily digested and its digestion products are absorbed in the upper GIT. However, a minor fraction of starch reaches the lower GIT and is referred to as ‘resistant’ [9]. RS covers a wide range of materials and can be subdivided, i.e., RS I through RS IV [59]. An additional type of RS, RS V, is often included in this classification. RS I is resistant to digestion as it is locked within the storage cell. RS II comprises native starch granules and is poorly digested by human digestive enzymes due to its conformation and semi-crystalline structure. Type III of RS is retrograded starch. A group of chemically modified starches that are etherized, esterified or cross-linked to chemicals is classified under RS IV. The digestibility of this type of RS is reduced due to their chemical modification. RS V is a group of RS representing amylose-lipid complexes that are usually formed from high amylose starches. The polysaccharide fraction of RS V was found to be water insoluble and unsusceptible to degradation by α-amylase [59,60]. RS as a functional fiber, plays a crucial role in intestinal physiology. Similarly to FOS, RS offers fermentable carbohydrates to colonic bacteria and, as such, serves as a growth substrate for probiotic bacteria [7]. RS demonstrates its physiological effects on the human colon mostly through production of SCFA, lowering the colonic pH and reducing the ammonia, phenols and secondary bile acids concentration in the colon [14]. RS is widely used as encapsulation material for probiotics in order to enhance their stability against severe environmental factors such as low gastric pH [59]. The protecting effect of three complementary prebiotics were compared through encapsulation of *Lactobacillus acidophilus* spp. in yoghurt (in vitro acidic condition). In comparison to Raftiline^®^ and Raftilose^®^, Hi-maize^®^ (a commercial high amylose maize starch) provided the highest degree of protection to the encapsulated bacteria after 3 h of incubation at pH 2.0 [61]. Moreover, there is an emerging trend towards creating RS in food products to benefit from its physiological effects through lowering the energy amount and bio-accessible carbohydrate content. Further studies are under investigation with respect to RS’ potential to escalate the initiation of satiety and to reduce the glycemic index [62].

## 3. Comparison of DF in Different Wheat Grains

Both genotype and environment have been reported to affect the content of DF and its composition in wheat. In order to observe the contribution of these two factors to DF properties, different spring and winter varieties of wheat were compared in a detailed field experiment by Gebruers et al. [13]. Their results showed that the variability in the level of total DF, total NSP and total AX were similarly affected by both genotype and environment. They concluded that wheat varieties, possessing a high level of DF, are great ingredients for producing healthy or health-boosting food products. These products could then simultaneously have a high level of total DF, but also display a high level of soluble DF and/or prebiotic oligosaccharides made in situ through enzyme activities in the GIT. They also suggested that as the levels of DF are notably affected by genotype, carefully selecting wheat varieties may play a role in the health and functional properties of wheat. A considerable effect of genotype allows breeders to evolve steady varieties with higher or lower contents of DF components keeping in mind health or technological demands [13].

Wheat kernels, and especially ancient ones, have been formerly introduced as a rich source of health-boosting compounds [14]. In a study by Ficco et al. [9], the level of potentially prebiotic polysaccharides including RS and other DF, were evaluated in ancient and modern durum wheat varieties (*Triticum turgidum* ssp. genotypes). In fact, two groups of genetic materials were classified. Group (1): nine ancient genotypes including landraces and obsolete cultivars and group (2): three modern genotypes including two semi-dwarf cultivars and one breeding line, derived from an “old × modern” cross, were tested. Although the level of the total starch was higher in modern wholemeal than ancient wholemeal, the opposite trend was reported for RS. Moreover, the RS detected in wholemeal was classified under RS I and RS II types. The difference in DF content was significantly correlated with the genotypes. However, no clear trend in total DF was observed between the two groups of wheat [9]. In this study, the facility and milling conditions for all the grains including ancient and modern were the same; hence, the difference in RS level between ancient and modern genotypes was associated to the evolution of the modern grains which might have altered the granular structure and/or the endosperm cell walls. Nevertheless, these two genotypes of durum wheat had been grown under different conditions [9]. Shewry and Hey [63] also reported that ancient wheat species einkorn, emmer, spelt and Khorasan are slightly different from modern wheat species (common and durum) regarding the levels of most bioactive compound and DF [63]. The values of DF collected by them through browsing the literature data showed that the ancient wheats have a tendency to be lower in amount of DF than those found for common wheats in the same studies (Table 3). This suggestion that old wheats are healthier than modern wheats is not supported by this report [63]. However, DF content is not the only factor that can be taken to account for defining the health benefits of the wheat and certainly there are other nutritional components that need to be considered. Moreover, further studies are needed to deeply compare multiple genotypes of old and modern wheat grown in replicate multi-side field experiments.

DF extracted from modern and ancient wheat varieties have been also used as prebiotic source to compare their effect on the activity of the probiotics. For instance, the soluble DF of a number of durum wheat grains including modern and ancient were identified as potential prebiotics that selectively cause the in vitro reproduction of *Bifidobacterium pseudocatenulatum* B7003 and *Lactobacillus plantarum* L12 [14]. Soluble DF extracted from Solex, as a modern variety, and from Kamut^®^ Khorasan, a trademark of ancient wheat cultivar Khorasan, were reported to show the highest efficacy in promoting the tested probiotic strains in the GIT [14]. Nonetheless, as the fermentation of the soluble DF in the GIT is part of a cooperative process, further studies are required to consider the effects of other cooperators, e.g., cross-feeding between host microflora and also nutrient competition, on the viability, proliferation and activity of the beneficial bacteria in the colon [14]. Bordoni et al. [64] reported the health-prompting properties of Kamut^®^ Khorasan as well. Based on their study, this ancient wheat has the potential to be used as a raw ingredient for amending the prebiotic properties of wheat products. However, they did not establish whether this health benefits are associated to this ancient wheat in particular or to all the ancient grains in general.

## 4. Boosting Prebiotic Potential of Cereal Components

Cereal components may naturally contain prebiotics which promote the probiotic growth. With regard to wheat, and taking into account that DF is concentrated in the outer layers of the kernel (bran), producing whole-grain flour is one of the options in order to provide the daily diet with a high DF source. In addition, as previously explained, immature wheat kernels could also be a great choice for providing a high degree of DF, and as a result, possibly enhanced prebiotic properties compared to the mature wheat kernels. However, the prebiotic potential of cereal components could also be improved through kernel and flour/wholemeal pre-processing techniques. Certain techniques including pre-fermentation and sprouting have been reported to affect the prebiotic properties of cereal grains. Fermentation is a relatively popular ancient technique of food preservation. Recently, there has been renewed interest in fermentation as it presents opportunities for improving the nutritional and functional properties of food. For instance, sourdough fermentation products affect the intestinal health through several possible mechanisms including changing the DF population and its fermentability, production of exopolysaccharides and oligosaccharides possessing prebiotic potential and/or producing secondary metabolites that affect the gut bacteria [3]. The fermentation of wheat wholemeal-based sourdough products was reported to increase the solubility of wheat bran AX [65]. Therefore, sourdough fermentation might affect the prebiotic properties of AX by providing more accessible AX for the growth of intestinal beneficial bacteria. Pre-fermentation of bran with yeast or, more in particular, with yeast and lactic acid bacteria might be a simple tool for increasing the prebiotic properties of cereal components. The effect of fermentation is assumed to be brought about by enzyme activity during this process as well as by the change of pH that will alter the fermentation pattern of DF. In a study by Napolitano et al. [8], Trichoderma hydrolytic enzymes have been successfully used in converting the insoluble DF of the durum wheat to soluble DF showing prebiotic potential. In this study, enzymatic treatments of insoluble DF resulted in generation of soluble DF that supported the growth of bifidobacteria and lactobacilli in a gut model.

Sprouting or germination is another ancient traditional kernel pre-processing technique that could be considered as a green tool for amelioration of cereal health properties. It was reported that sprouted durum wheat cultivars in an in vitro digestion model significantly increased the prebiotic index (prebiotic activity score) [66]. However, this increase was wheat genotype dependent. The effect of sprouting on the prebiotic index has been associated to the change of nutritional properties [66]. In a study on wheat kernels (cultivar Tommi), a substantial increase of total and soluble DF was observed in prolonged sprouting up to 168 h; while the level of insoluble DF was decreased by half [67]. Potential prebiotic activity of sprouted rye was determined in an in vitro study by Noori et al. [3]. Their results showed that there is a positive correlation between the concentration of sprouted rye extract and the viability of *Lactobacillus acidophulis* and *Bifidobacterium animalis*.

## 5. Isolation of Cereal DF

It is clear that cereals and cereal DF function as prebiotic food ingredients. The isolation of prebiotic fractions of various cereal grains and even cereal by-products (e.g., bran or brewer’s spent grain) seems propitious. The isolation of these fractions can be conventionally initiated via physical processing technologies (e.g., pearling, milling and sieving,) [7]. The selection of the most appropriate mechanical processing technology for the separation of a specific DF fraction is based on the distribution of the specific fiber in the kernel and kernel morphology [7]. The isolation and purification of DF is typically performed based on the conventional milling process. Combining pearling processing with classical milling produces streams of specific components including the outer or inner pericarp, the seed coat, the aleurone layer, the germ and the starchy endosperm. In a next step, each stream can be further purified to target a particular DF [7]. As previously mentioned, pearling technology allows isolating β-glucan enriched wheat-derived fractions, although the wheat kernel does not carry a high level of β-glucan. Wheat pericarp, seed coat tissue and the nucellar epidermis collectively make a rich source of AX and lignin. The bran segment of the crease plus the sub-aleurone fraction are segregated by milling and consequently form a rich source of β-glucan in combination with the aleurone section derived from pearling [7]. In order to effectively apply the pearling technology to other cereal kernels, appropriate conditioning of the kernels before pearling and also the design of the debranner need to be considered [7].

Thermal (e.g., using hot or boiling water) and chemical (e.g., acid or alkaline hydrolysis) treatments are broadly used for DF extraction [68]. Using boiling water to extract carbohydrates is one of the most commonly used and low-cost techniques. Nevertheless, in comparison with chemical extraction, the eventual carbohydrate yield is relatively low [68]. Chemical treatments involving acid or alkaline hydrolysis have been extensively used to isolate DF, both by itself or in combination with thermal processing [68]. Although chemical treatments are relatively popular, the long processing time, high temperatures and the possibility of chemical-induced modifications of functional groups on the extracted DF are counted as the main disadvantages of this technique [68].

Nowadays, enhanced, efficient innovative techniques have been introduced as alternatives for the above conventional extraction methods in order to improve the extraction process and reduce the time, temperature and/or the use of acid or alkaline solvents, hence saving energy, resources and costs [68,69]. In this regard, enzyme-assisted extraction as an emerging approach has been used separately or in combination with conventional methods in order to isolate or favor the purification of specific DF [68,70]. In addition, the hydrolytic activity of the enzymes might result in converting the insoluble DF to soluble DF. However, the enzymatic hydrolysis pattern and resulting products depend on the type and selectivity of the enzyme as well as the process conditions [71]. In a study by Lin et al. [72], the insoluble and soluble DF of wheat bran were isolated by enzymatic hydrolysis using amylases, glucosidases and proteases. Nonetheless, the long reaction time and the high cost of enzymes are counted as disadvantages of the enzymatic treatments to isolate DF [73].

Moreover, emerging technologies (e.g., ultrasound, microwave and high-pressure processing) have been directed successfully toward the extraction of DF [68]. Ultrasonically assisted extraction (UAE) has been used over recent years for extraction of fibers [74]. This extraction technique employs ultrasound waves of high intensity with a frequency exceeding 20 kHz. UAE breaks the cell walls, and as such, results in the release of the cell walls components in the solvent [70,74]. In comparison to other extraction techniques, UAE takes less time and needs lower temperature [74]. Indeed, this technology expedites the transfer of mass and improves the efficiency of the extraction through reduction of the extraction time and the energy cost [69,75]. Additionally, UAE allows controlling molecular weight of the isolated fibers [74]. The mentioned advantages of UAE make it a great choice for achieving not only a higher extraction yield but also a greater purity of the isolated polysaccharides [69]. Nevertheless, the high intensity of the ultrasound waves is also counted as one of the disadvantages of this technique as it might break the polymer structure and destroy the polysaccharides’ integrity [70]. This method was used in a study by Reis et al. [76] in order to produce starch-free AX-rich extracts, with enhanced prebiotic properties, from brewer’s spent grain. The results showed that the ultrasound processing significantly reduced the energy consumption and extraction time (from 7 h to 25 min) relative to the conventional alkaline hydrolysis, for extracting a comparable amount of AX.

Microwave assisted extraction (MAE) is a more recently explored method than UAE for DF isolation [75]. Microwaves are a type of electromagnetic radiation with a frequency of 300 MHz–300 GHz [75]. The energy of the microwaves heats the solvent and results in separation and release of the compounds of interest from the sample matrix in the solvent phase [70]. In comparison to thermal extraction, MAE conducts the heat throughout the sample volume, while the conventional thermal extraction heats the sample from the outside to the inside [75]. This technique was previously used to extract the AX and AX-oligosaccharides from brewer’s spent grain [77]. The extraction of the target compounds increased with the increase of the temperature and different sequential MAE steps resulted in separation of the fractions rich in AX, AX-oligosaccharides and feruloylated AX-oligosaccharides [77].

High hydrostatic pressure (HHP) or high-pressure processing has been extensively used over the last decades for the extraction of bioactive compounds. It has been proven that this technology modifies the soluble DF/insoluble DF ratio [74]. In HHP treatments, a high pressure is exerted on a sample ranging from 100 to 1000 MPa. The HHP can be combined with thermal processing [68,74]. Isostatic pressure is applied throughout the whole sample, and therefore, all the molecules experience an equal pressure [74]. The application of HHP for DF extraction is currently mainly studied for fruits and vegetables and their by-products such as the peels of orange, mango and prickly pear and also purple fleshed potatoes [78,79]. As another example, applying the combination of HHP and controlled thermal treatments resulted in a higher soluble DF/total DF ratio in okara, the main by-product of soymilk and tofu production which is rich in insoluble DF [80]. Further studies on the use of HHP for extraction of DF from cereal sources could improve our knowledge and provide possible new industrial applications for cereal prebiotics.

Blasting extrusion processing is also a relatively novel technique that can be used to increase the soluble DF content and extractability of soluble DF polysaccharides [81]. In this process, the combination of high temperature, pressure (10–50 MPa) and shear are applied to the material in the extrusion unit. This material could be wheat bran. In brief, the extrusion unit is fed by the wheat bran and a semi-fluid is formed under high temperature and high pressure. The flow stream as a result of the shear force, originated from the Archimedes-type screw, reaches the die nozzle. After the sudden release of the pressure, the DF structures are partially disrupted and soluble DF are formed [81]. This technique was used to successfully increase the content of soluble DF of wheat bran and isolation of a soluble polysaccharide consisting of mostly glucose and xylose and to some extent arabinose and galactose [81].

## 6. Future Perspective

The information provided in this review considers the potentially prebiotic components of cereals and their effects on probiotic growth, based on in vitro and/or in vivo studies. However, new studies that pursue the food industrial application of these prebiotics are urgently needed. Further research is required to shed light on the specific effect and mechanism of cereal prebiotics and put this in a wider perspective to acknowledge the complexity of food. Examples of avenues to study are the effect of combining prebiotic molecules, the result of interaction with other food components on the prebiotic potential and the overall metabolic effects. Although isolation of a specific DF from various cereals or cereal by-products along with its incorporation in food industry seems promising, it should be noted that the extraction may be costly, generates new side-products, and the processing conditions could notably impact the DF properties and the prebiotic potential. As such, more effort should be devoted to studying more complex matrices that are better reflecting cereal prebiotic source materials (e.g., bran, brewer’s spent grain, …). In addition, cereal biopolymers are not only appropriate substrates for the growth of probiotics, but are also suitable carriers for delivery of probiotics to the human GIT. The latter is a relatively new area of research but has great potential for future functional food development.

## Figures and Tables

**Table 1:** Total dietary fiber content in cereal wholegrains [7].

Cereal Type	Rye	Corn	Triticale	Oats	Wheat	Sorghum	Barley	Finger Millet	Rice
Total DF (% dry basis)	15.5	15	14.5	14	12	10.7	10	6.2–7.2	3.9

**Table 2:** Fructan level (% dry basis) in wholegrains of different cereals.

Cereals	Fructan Level (% d.b.)	Number of Tested Samples	References
Rye	5.7 & 5.1 4.6–6.6 3.6–4.6	2 13 18	[42] [43] [44]
Wheat	0.7–2.9 1.5–2.3 1.7 & 2.4	43 19 2	[45] [45] [42]
Barley	0.9–4.2 1.9 & 2.3	20 2	[46] [42]
Oats	Traces-0.2 1.0	121 2	[47] [42]

**Table 3:** Dietary fiber content (% dry basis) of modern (common and durum) and ancient (spelt, emmer and einkorn) wheats [63].

	Common	Spelt	Emmer	Einkorn	Durum
Total dietary fiber Mean Range Number of samples	14.96 11.3–21.5 168	11.18 8.8–14.9 54	9.2 7.2–12.0 8	10.8 8.7–16.7 21	13.1 10.7–15.5 13
Insoluble dietary fiber Mean Range Number of samples	11.3 9.8–13.2 11	9.6 7.8–12.9 21		6.9 1	10.6 9.5–11.7 2
Soluble dietary fiber Mean Range Number of samples	1.7 1.4–2.2 6	1.6 0.8–2.5 26		1.7 1	1.6 1.6 2
Total arabinoxylans Mean Range Number of samples	6.9 6.11–7.89 11	5.74 4.68–6.82 28			
β-glucans Mean Range Number of samples	0.72 0.37–0.95 167	0.64 0.23–0.90 42	0.36 0.3–0.4 8	0.39 0.25–0.48 20	0.37 0.25–0.53 11
